# Iron-induced kidney cell damage: insights into molecular mechanisms and potential diagnostic significance of urinary FTL

**DOI:** 10.3389/fmolb.2024.1352032

**Published:** 2024-02-21

**Authors:** Soraya Punchai, Nachayada Chaiyagot, Nadthanicha Artkaew, Apinya Jusakul, Ubon Cha’on, Raynoo Thanan, Kulthida Vaeteewoottacharn, Worachart Lert-Itthiporn

**Affiliations:** ^1^ Department of Biochemistry, Faculty of Medicine, Khon Kaen University, Khon Kaen, Thailand; ^2^ Chronic Kidney Disease Prevention in Northeastern Thailand, Khon Kaen University, Khon Kaen, Thailand; ^3^ Centre for Research and Development of Medical Diagnostic Laboratories, Faculty of Associated Medical Sciences, Khon Kaen University, Khon Kaen, Thailand; ^4^ Center for Translational Medicine, Faculty of Medicine, Khon Kaen University, Khon Kaen, Thailand

**Keywords:** iron, ferritin light chain, ferroptosis, kidney cells, protein oxidation, cell death

## Abstract

**Background:** Iron overload can lead to organ and cell injuries. Although the mechanisms of iron-induced cell damage have been extensively studied using various cells, little is known about these processes in kidney cells.

**Methods:** In this study, we first examined the correlation between serum iron levels and kidney function. Subsequently, we investigated the molecular impact of excess iron on kidney cell lines, HEK293T and HK-2. The presence of the upregulated protein was further validated in urine.

**Results:** The results revealed that excess iron caused significant cell death accompanied by morphological changes. Transcriptomic analysis revealed an up-regulation of the ferroptosis pathway during iron treatment. This was confirmed by up-regulation of ferroptosis markers, ferritin light chain (FTL), and prostaglandin-endoperoxide synthase 2 (PTGS2), and down-regulation of acyl-CoA synthetase long-chain family member 4 (ACSL4) and glutathione peroxidase 4 (GPX4) using real-time PCR and Western blotting. In addition, excess iron treatment enhanced protein and lipid oxidation. Supportively, an inverse correlation between urinary FTL protein level and kidney function was observed.

**Conclusion:** These findings suggest that excess iron disrupts cellular homeostasis and affects key proteins involved in kidney cell death. Our study demonstrated that high iron levels caused kidney cell damage. Additionally, urinary FTL might be a useful biomarker to detect kidney damage caused by iron toxicity. Our study also provided insights into the molecular mechanisms of iron-induced kidney injury, discussing several potential targets for future interventions.

## 1 Introduction

Iron is an essential element required for numerous biological processes in the human body, including oxygen transport, energy production, and enzymatic reactions (Abbaspour et al., 2014). However, an excess of iron accumulation due to disrupted iron homeostasis gives rise to a condition termed iron overload. This condition may manifest in different scenarios, including hereditary hemochromatosis, regular blood transfusions, and acute iron poisoning (Kohgo et al., 2008; Zekavat et al., 2023). It is characterized by abnormal iron deposition in multiple organs, including the liver, heart, and bone, leading to tissue damage and dysfunctions (Kohgo et al., 2008; Naito et al., 2015; Rios-Silva et al., 2023). The kidney is particularly susceptible to iron overload-induced injury especially when patients received intravenous iron supplement (Naito et al., 2015; Rios-Silva et al., 2023). Iron accumulation in the kidneys disrupts normal physiological functions and contributes to the development of kidney diseases (Zumbrennen-Bullough and Babitt, 2014). The growing concern over cases of iron overload and associated renal impairment underscores the need for understanding the processes of iron-induced kidney disease (Wojtaszek et al., 2020; Zheng et al., 2023).

Cell death is a fundamental biological process that occurs in both physiological and pathological conditions (Fink and Cookson, 2005; Ai et al., 2024). Multiple forms of cell death have been identified, each of which is characterized by distinct morphological and biochemical features (Liu et al., 2023). Apoptosis, necrosis, autophagy, and ferroptosis represent distinct modes of cell death. Apoptosis, characterized by programmed cell death, involves controlled cellular dismantling and is crucial for tissue development and homeostasis (Fink and Cookson, 2005; Cui et al., 2021). In contrast, necrosis is an uncontrolled form of cell death associated with rapid plasma membrane rupture and the release of intracellular contents, often triggering an inflammatory response (Cui et al., 2021). Autophagy is a process that facilitates the degradation and recycling of cellular components to maintain cellular homeostasis during stress conditions (Cui et al., 2021). Ferroptosis, a recently described form of regulated cell death, is characterized by iron-dependent lipid peroxidation and subsequent lethal accumulation of lipid reactive oxygen species (ROS) (Li et al., 2022; Zheng et al., 2023).

Kidney diseases, including acute kidney injury (AKI) and chronic kidney disease (CKD), are common and significant public health concerns (Iroegbu et al., 2022). These conditions affect millions of people worldwide and lead to kidney cell death and dysfunction (Iroegbu et al., 2022). Thus, obtaining a better understanding of the role iron plays in these diseases will help develop targeted therapies (Li et al., 2022; Zhang et al., 2022). Moreover, research into iron-associated cell death will provide insights into slowing or preventing kidney disease progression (Li et al., 2022; Zhuo et al., 2022). In this study, we aimed to investigate the effect of iron overload on kidney cells and explore the specific involvement of various forms of cell death, with a particular focus on ferroptosis. By unraveling the molecular pathways underlying iron-induced kidney injury and ferroptosis, we aspire to contribute to the understanding of the pathophysiology of iron overload-related kidney diseases. Furthermore, our findings may pave the way for the development of novel therapeutic strategies to prevent or mitigate iron-induced renal damage. Through this study, we sought to advance our knowledge of iron overload-related kidney diseases and provide potential avenues for targeted interventions, ultimately improving patients’ outcomes and quality of life.

## 2 Materials and methods

### 2.1 Study subjects

This study used the setting and the subjects from the CKD screening project of the Chronic Kidney Disease in the Northeast Thailand project, as described elsewhere (Cha’on et al., 2020). Briefly, residents of Koksamran Village, located in the rural area of Northeast Thailand, were screened for CKD. Blood and urine samples were collected from the participants. The samples and data from a total of 376 people were included in this study. Approval for the sample collection was obtained from the Institutional Human Ethics Review Board of Khon Kaen University (HE661039).

### 2.2 Measurement of protein in serum and urine

The serum was sent to the clinical chemistry laboratory at Srinagarind Hospital, Khon Kaen, Thailand. The estimated glomerular filtration rate (eGFR) was calculated based on the CKD-EPI equation (Levey et al., 2009). Urine samples were stored at −80°C before protein detection. We developed an in-house indirect enzyme-linked immunosorbent assay (ELISA) to detect ferritin protein in urine. Urine from a random selection of 148 samples, diluted at 1:10, was plated overnight at 4°C on 96-well flat-bottom, high-binding plates without lids (Corning, United States of America). After blocking with 5% skimmed milk, the primary antibody, diluted at 1:500 anti-ferritin light chain rabbit monoclonal antibody (A11241, Abclonal, MA, United States of America), was added and incubated for 1 h at 37°C. After washing, the secondary antibody (Anti-rabbit IgG, HRP-linked antibody; diluted at 1:500) (7074S, ABclonal, MA, United States of America) was added and incubated again at 37°C for 1 h. O-phenylenediamine dihydrochloride (OPD) (Thermo Scientific) was added to each well. The optical density (OD) of the wells was read at the wavelength of 492 nm using a microplate reader (Sunrise, Tecan Trading AG, Switzerland).

### 2.3 Cell culture

Human kidney cell lines, HEK293T and HK-2, from the American Type Culture Collection (ATCC), were used in this study. Approval for this experiment was obtained from the Institutional Human Ethics Review Board of Khon Kaen University (HE651422). HEK293T and HK-2 cell lines were cultured in Dulbecco’s Modified Eagle’s Medium (DMEM) (Gibco, Life Technologies; Grand Island, NY, United States of America) supplemented with 10% fetal bovine serum (HyClone Laboratories, Inc.; Logan, UT, United States of America) and 1% antibiotic antimycotic reagent (Invitrogen) in a 5% CO_2_ incubator at 37°C. *Mycoplasma* contamination was routinely checked. The effects of ferric ammonium citrate (FAC), (SIA-F5879-100G, Sigma-Aldrich Co., Missouri, United States of America) treatment on those cell lines were assessed using a cell viability test.

### 2.4 Cell viability

To determine the cell number, the sulforhodamine B (SRB) assay was performed (Sigma-Aldrich Co., Missouri, United States of America, Lot No. MKBQ6027V). HEK293T cells were plated at a density of 2 × 10^4^ cells/well, while HK-2 cells were plated at a density of 7.5 × 10^3^ cells/well in 96-well plates with 90 μL of medium. After 24 h of incubation, the cells were treated with varying concentrations of FAC (1, 2.5, 5, and 10 μM) combined with an iron carrier, 8-hydroxyquinoline (8-HQ), (Sigma-Aldrich Co., Missouri, United States of America, catalog No. 252565), at a concentration of 5 μM for HEK293T and 10 μM for HK-2 cell lines. The concentration of 8-HQ was determined to be non-toxic to the cells, as assessed by both cell morphology and cell viability. The half-maximal inhibitory concentration (IC50) of FAC was determined from the dose-response curve (Supplementary Figure S1). Subsequently, the cells were incubated for an additional 24 h. After treatment, the cells were fixed with cold 10% trichloroacetic acid (TCA) (Lot No. 2009272) and stored at 4°C for 1 h. They were then washed with distilled water and stained with 0.4% SRB in 1% acetic acid for 30 min at room temperature in the dark. After washing with 1% acetic acid, the cells were solubilized in 10 mM Tris-base (pH 10.5) for 20 min on a shaker in the dark. The absorbance of the solubilized cells was measured at 540 nm using a spectrometer (Sunrise, Tecan Trading AG, Switzerland) to quantify the cell number.

### 2.5 Apoptosis analysis

Apoptosis of HEK293T and HK-2 cells was assessed using an Annexin V pacific blue/propidium iodide (PI) staining. The cells were cultured in a 6-well plate at a density of 6 × 10^5^ cells/mL for HEK293T and 2 × 10^5^ cells/mL for HK-2 cells. For one group of each cell type, pre-treatment or post-treatment with deferoxamine (DFO) (Sigma-Aldrich Co., Missouri, United States of America, Catalog No. 138-14-7) at a concentration of 10 µM was carried out for 2 h before or after exposure to FAC. The cells were treated with FAC for 24 h, washed with phosphate buffer solution (PBS), and stained with Annexin V pacific blue (BIOL-640918, Biolegend) for 15 min in the dark, then stained with PI (Invitrogen) before detection. This staining method allowed the detection and quantification of apoptotic cells by flow cytometry using a BD FACSCanto™ II Cell Analyzer (BD Biosciences; Franklin Lakes, NJ, United States of America). Data were analyzed with FlowJo™ software version 10.4 (Tree Star Inc; Ashland, OR, United States of America).

### 2.6 Mitochondrial morphology

The ultrastructural changes associated with iron-induced ferroptosis, with special attention on mitochondrial morphology, were assessed using transmission electron microscopy (TEM). HEK293T and HK-2 cells were grown to confluency on 6-cm dishes and treated with FAC for 24 h. After treatment, the cells were washed with PBS and fixed with 2.5% glutaraldehyde in 0.1 M sodium cacodylate buffer (pH 7.4) at 4°C for 24 h. Then, the cells were fixed with 1% osmium tetroxide, dehydrated in an ethanol series, and embedded in Spurr’s resin. Ultrathin sections (70–90 nm) were cut using an ultramicrotome, placed on copper grids, and stained with uranyl acetate and lead citrate. The sections were then examined using a JEOL JEM-1400 Plus TEM operated at 200 kV. TEM images were captured with a Gatan Orius SC1000 CCD camera and analyzed using Gatan Digital Micrograph software. At least 10 random fields of view were imaged per sample, and the images were evaluated by two independent observers to ensure consistency of the results.

### 2.7 Measurement of carbonylated protein

As a major hallmark of oxidative stress-related disorders, protein carbonylation in the iron-treated cells was examined. For this purpose, cells were treated with varying concentrations of FAC combined with 8-HQ for 24 h. Pierce™ bicinchoninic acid (BCA) Protein Assay Kit (Thermo Fisher Scientific, Lot No) was used to quantify the total protein level. To quantify protein carbonylation, the 2,4-dinitrophenylhydrazine (DNPH) assay (Sigma-Aldrich Co. United States of America, Lot No.) was employed, and the complex of protein carbonyl groups with DNPH was spectrophotometrically detected at 373 nm using a spectrometer (Sunrise, Tecan Trading AG, Switzerland). The measurements were performed in three independent experiments.

### 2.8 Measurement of malonaldehyde (MDA)

As a marker for oxidative stress induced by excess iron, malonaldehyde (MDA) in the cells treated with/without FAC for 24 h was quantitatively measured using a lipid peroxidation assay kit from Sigma-Aldrich (Sigma-Aldrich Co., Missouri, United States of America, Lot No. 8L03K07390). The assay involved the reaction of MDA with thiobarbituric acid (TBA) to form a colored complex, which was quantified by measuring the absorbance at 532 nm using a spectrometer (Sunrise, Tecan Trading AG, Switzerland). MDA concentrations were determined using a standard curve.

### 2.9 RNA sequencing

For RNA sequencing, HEK293T cell lines were seeded in 6-cm dishes and treated with 2.5 μM of FAC and 5 μM of 8-HQ. RNA was then extracted using the RNeasy Mini Kit from Qiagen (Lot No.172042559). The quality of the extracted RNA was assessed using a Qubit fluorometer. RNA with specific purity and integrity criteria was submitted to a public company for pair-ended sequencing on an Illumina NovaSeq 6000 system. The processes were performed in triplicate to ensure accurate results. The sequencing data were uploaded to the Galaxy online platform, and we performed data analysis using the public server at usegalaxy.org (Community, 2022). The criteria to determine the cut point for differentially expressed genes were *p-value* < 0.001 and log_2_|fold change| > 0.05. Pathway analysis was performed using the KEGG database in DAVID (Kanehisa and Goto, 2000; Dennis et al., 2003).

### 2.10 Western blotting analysis

To determine protein expression levels, SDS-PAGE and Western blotting were performed. Cell lysates were prepared using lysis buffer and centrifuged to collect the supernatant. Protein concentration was determined using the Bradford assay. Samples were treated with sample buffer and subjected to SDS-polyacrylamide gel electrophoresis. Proteins were transferred to a PVDF membrane and blocked with skim milk. Subsequently, the membranes were incubated with specific antibodies, followed by secondary antibodies. The immunoreactivity was detected using ECL TM Prime Western blotting Detection (ImageQuant LAS800 system, GE Healthcare Japan, Tokyo, Japan). Signal intensities were analyzed using ImageJ software (ImageJ, U. S. National Institutes of Health, Bethesda, Maryland, United States of America).

### 2.11 Real-time quantitative PCR

FTL, PTGS2, GPX4, and ACSL4 mRNA expression levels in FAC-treated kidney cell lines were determined by real-time RT-PCR. Beta-actin was used as an internal control. The mRNA expression levels were measured using the LightCycler^®^ 480 real-time PCR system (Roche Diagnostics, Mannheim, Germany). For each PCR condition, SYBR Green I Master (Roche Diagnostics, Mannheim, Germany) and the following primers were used: FTL: 5′-TAC​GAG​CGT​CTC​CTG​AAG​ATG​C-3′ and 5′-GGT​TCA​GCT​TTT​TCT​CCA​GGG​C-3′, PTGS2: 5′- ATA​TGT​TCT​CCT​GCC​TAC​TGG​AA -3′ and 5′-GCC​CTT​CAC​GTT​ATT​GCA​GAT​G-3′, ACSL4: 5′- TGG​AAG​TCC​ATA​TCG​CTC​TGT -3′ and 5′- TTG​GAT​ACA​GCA​TGG​TCA​AA-3′, HMOX-1: 5′- CAA​CAT​CCA​GCT​CTT​TGA​GGA -3′ and 5′- T GGG​CAG​AAT​CTT​GCA​CTT​TG-3′, Nrf-2: 5′- TAC​TCC​CAG​GTT​GCC​CAC​A-3′ and 5′- CAT​CTA​CAA​ACG​GGA​ATG​TCT​GC-3′, GSTP1: 5′- TAC​ACC​AAC​TAT​GAG​GCG​GG-3′ and 5′- AGC​GAA​GGA​GAT​CTG​GTC​TC-3’. Amplification was performed through a series of cycles. Duplicate samples were prepared and mean and SD values of cycle threshold (Ct) and melting temperature (Tm) were calculated. Gene expression levels were determined using the 2^^(-∆∆CT)^ method, where ∆CT = Ct, target-control.

### 2.12 Statistical analysis

Data were shown as the mean ± standard deviation (SD). The statistical significance of differences observed between the control group and the experimental groups was determined using the Student’s t-test. A value of *p* < 0.05 was considered significant. The data were analyzed using the GraphPad Prism^®^ 7.04 software (GraphPad Software Inc.; San Diego, CA, United States of America).

## 3 Results

### 3.1 Interplay between body iron markers and kidney function

**FIGURE 1:**
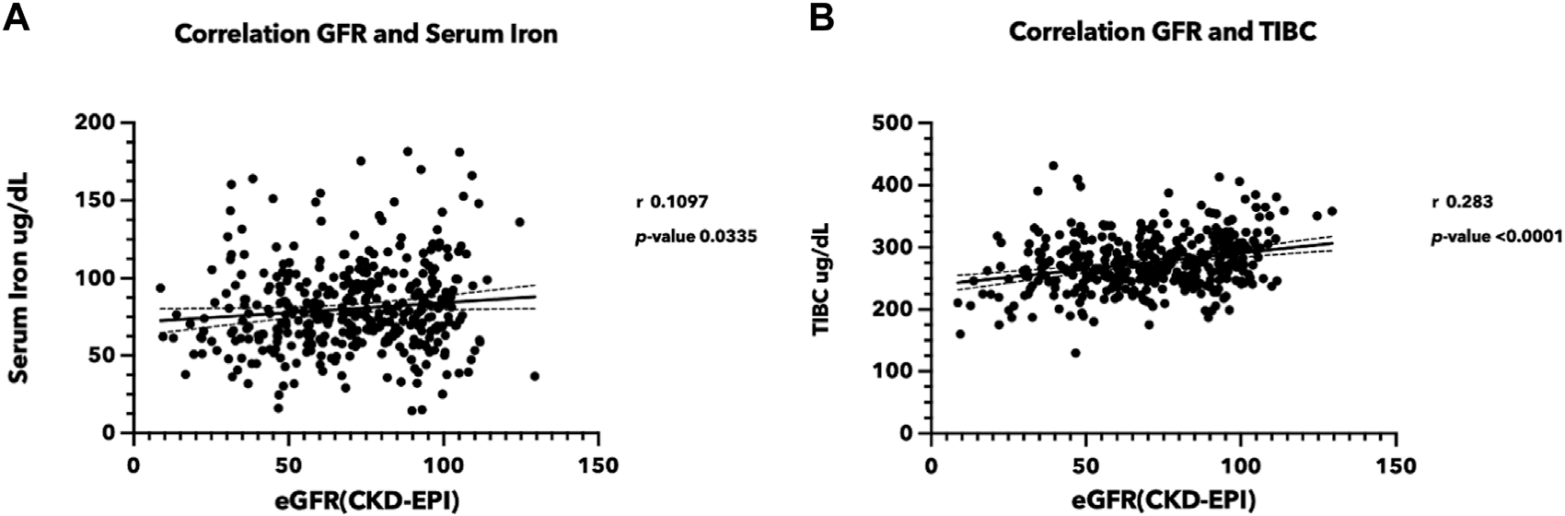
Correlation between iron status and estimated glomerular filtration rate (eGFR) **(A)** Correlation between serum iron level and eGFR, **(B)** between TIBC and eGFR. Serum iron, TIBC, and blood creatinine were measured in the sera from 376 volunteers participating in the Chronic Kidney Disease in Northeast Thailand project (Chaon et al., 2020). The eGFR was estimated using the CKD-EPI equation (Levey et al., 2009). (TIBC, Total iron binding capacity, eGFR: estimated glomerular filtration rate).

A total of 376 volunteers from the rural area of Northeast Thailand participated in this study (Supplementary Table S1). The female-to-male ratio was 1.88, and the average age of the participants was 63.50 years (range: 25–80 years). Serum iron and eGFR appeared to be trending in the same direction, with a correlation coefficient of 0.1097 (*p-value* < 0.05) (Figure 1A). Total iron binding capacity (TIBC) was significantly correlated with eGFR, with a correlation coefficient of 0.283 (*p-value* < 0.05) (Figure 1B). Serum ferritin exhibited a slight negative association with kidney function, whereas transferrin saturation showed no correlation with eGFR (Supplementary Figure S2). These findings support the likelihood of renal damage from iron.

### 3.2 Iron overload induced kidney cell death

**FIGURE 2:**
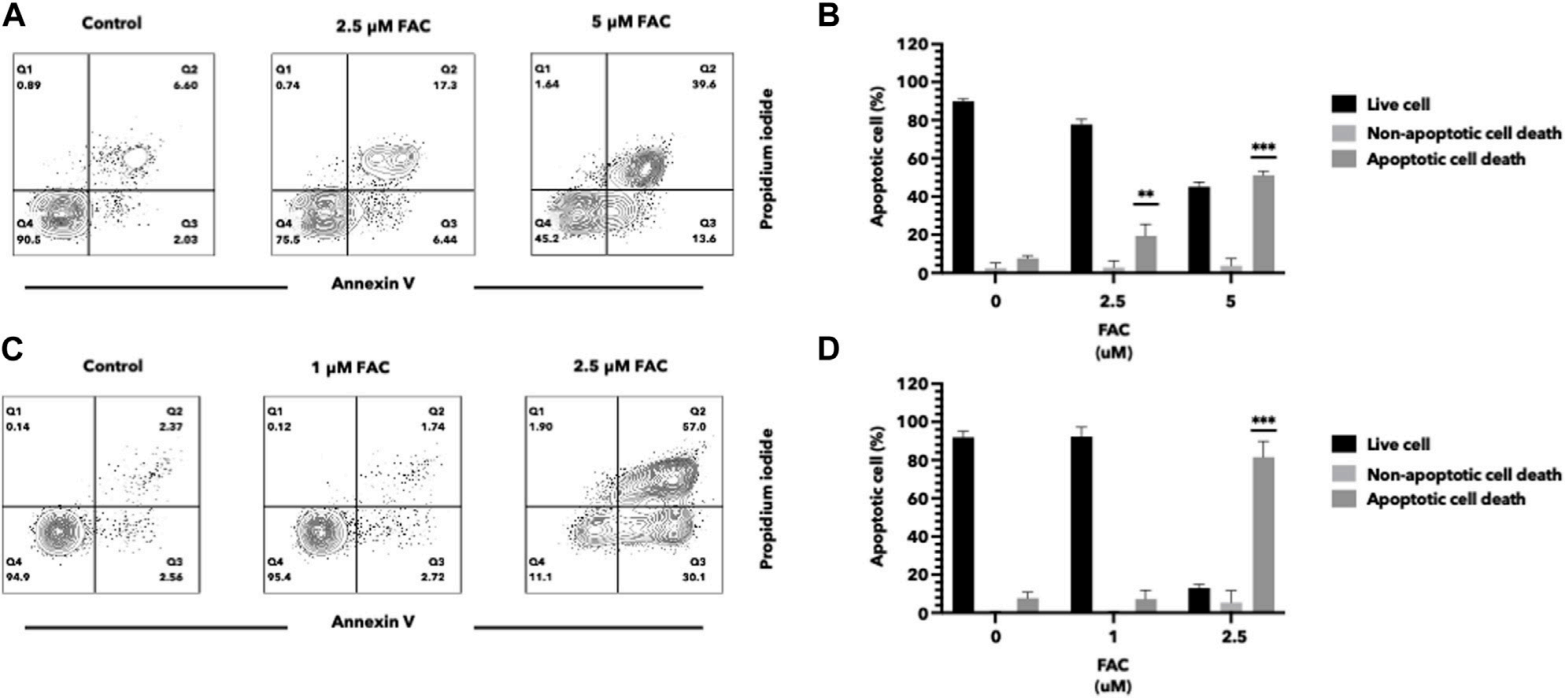
Iron induced cell death of HEK293T and HK-2 cell lines Effects of excess iron on the viability of kidney cell lines were determined by flow cytometry. HEK293T **(A,B)** and HK-2 **(C,D)** kidney cell lines were treated with FAC at 0, 2.5, and 5 μM, respectively. Contour plots of cells with annexin V and propidium iodide staining **(A,C)** were shown in four quartiles with the percentage of the cell number. (^*^ *p* < 0.05,^**^ *p* < 0.01, ^***^ *p* < 0.001, FAC, Ferric ammonium citrate).

Iron overload has been implicated in the induction of cell apoptosis in various cell types. In our study, we investigated the effect of iron overload on cell apoptosis in the HEK293T and HK-2 cell lines, representing human kidney cell lines. After treatment with an increasing dose of ferric ammonium citrate (FAC), we observed a significant increase in apoptotic cell death in both cell lines (Figure 2). To evaluate cell apoptosis, FAC-treated cells were stained with Annexin V/propidium iodide (PI) and analyzed by flow cytometry. The results showed a notable increase in the population of Annexin V-positive cells by FAC treatment, indicating the occurrence of early apoptotic events. Additionally, the population of PI-positive cells, representing late-stage apoptotic or necrotic cells, was also increased by FAC treatment compared to the control group.

### 3.3 Iron overload induced morphological changes in cells and their mitochondrial structure

**FIGURE 3:**
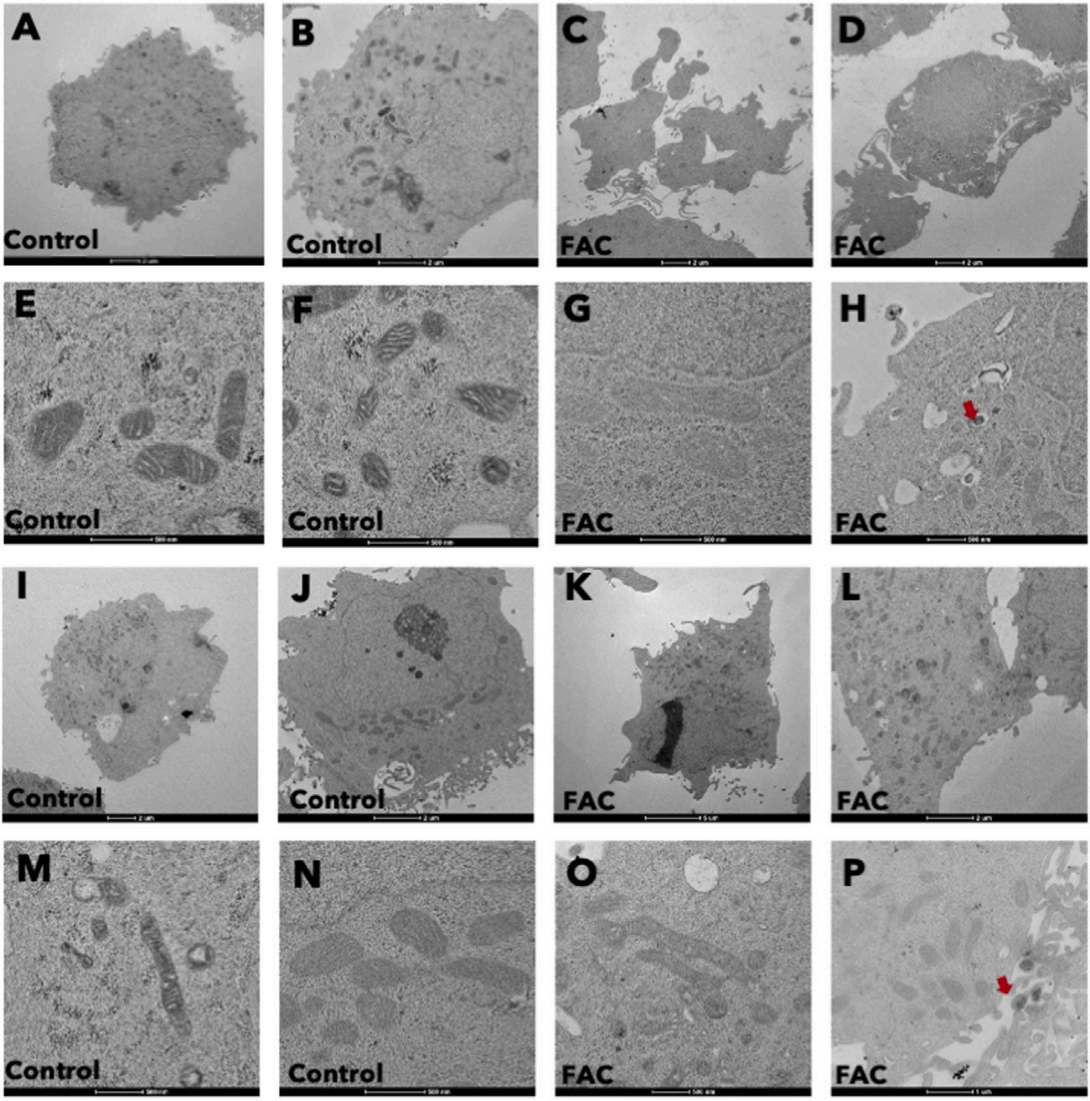
Iron caused morphological changes in cells and mitochondria of HEK293T and HK-2 cell lines Cell morphology of untreated control HEK293T cells **(A,B)** and of FAC-treated cells **(C,D)** showing cell swelling and membrane disruption. Mitochondrial morphology of control HEK293T cells **(E,F)** and FAC-treated cells **(G,H)** showing membrane rupture and remodeling. Cell morphology of control HK2 cells **(I,J)** and FAC-treated cells **(K,L)** showing cell swelling and membrane blebbing. Mitochondrial morphology of control HK-2 cells **(M,N)** and FAC-treated cells **(O,P)** showing a reduction in size with membrane rupture. Arrows indicate iron accumulation in the cells. (FAC, Ferric ammonium citrate).

Treatment of kidney cells with iron resulted in notable alterations in both cell and mitochondrial morphology, as evidenced by transmission electron microscopy (TEM) (Figures 3A–P). Specifically, cells treated with FAC displayed distinct morphological changes, including plasma membrane rupture and cell swelling. Also, the shape and size of mitochondria were affected, accompanied by mitochondrial membrane rupture and remodeling of cristae (Figures 3E, F, M, N). These morphological modifications were detected as early as 24 h after treatment with FAC at the IC50 concentration. These findings strongly suggest that iron treatment induces significant morphological alterations in both HEK293T and HK-2 cell lines, which reflects the cytotoxic effects of iron in the kidney.

### 3.4 Iron overload caused oxidative stress and ROS generation

**FIGURE 4:**
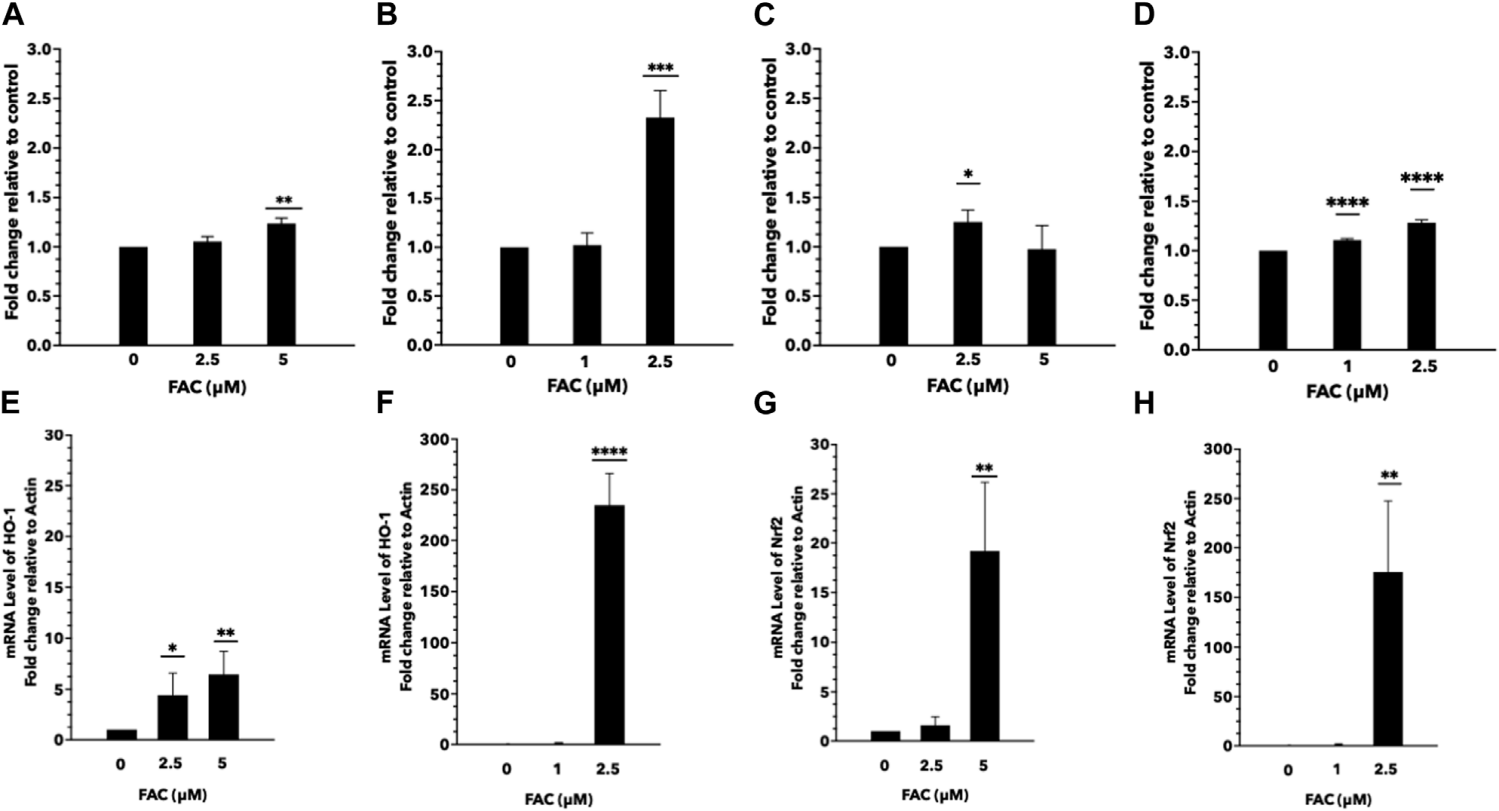
Excess iron causes oxidative stress and ROS generation in HEK293T and HK-2 cells Protein and lipid oxidation levels of HEK293T and HK-2 cells treated with various concentrations of FAC. Protein oxidation of HEK293T **(A)** and HK-2 **(B)**. Lipid peroxidation of HEK293T **(C)** and HK-2 **(D)**. The expression of oxidative stress-related genes. Nrf-2 expression in HEK293T **(E)** and HK-2 **(F)**. HMOX-1 expression in HEK293T **(G)** and HK-2 **(H)**. (^*^ *p* < 0.05,^**^ *p* < 0.01, ^***^ *p* < 0.001, ^****^ *p* < 0.0001, FAC: Ferric ammonium citrate, Nrf-2, nuclear factor erythroid 2-related factor 2, HMOX-1: heme oxygenase-1).

Iron overload is a known contributor to oxidative stress, characterized by an imbalance between the generation of reactive oxygen species (ROS) and the antioxidant defense system. In this study, we aimed to investigate the effects of iron overload on oxidative stress and ROS generation in the human kidney cell lines. To evaluate oxidative stress, we assessed lipid and protein oxidation as indicators of cellular damage. Lipid peroxidation was determined by measuring malondialdehyde (MDA) levels (Figures 4C, D), and protein oxidation was assessed by quantifying carbonyl group formation (Figures 4A, B). Our findings revealed a significant increase in both MDA levels and protein carbonylation in cells exposed to iron overload compared to the control group, indicating heightened oxidative damage. To gain insights into the underlying mechanisms of iron-induced oxidative stress, we examined the expression of oxidative stress markers, specifically nuclear factor erythroid 2-related factor 2 (Nrf-2) (Figures 4E, F) and heme oxygenase-1 (HMOX-1) (Figures 4G, H). Nrf-2 is a pivotal transcription factor involved in cellular antioxidant defense, while HMOX-1 is an enzyme induced under oxidative stress conditions. We observed an upregulation in the mRNA expression levels of Nrf-2 and HMOX-1 in both HEK293T and HK-2 cells exposed to iron (Figures 4E–H), indicating the activation of antioxidant response pathways in response to increased oxidative stress. These results provide evidence that iron overload induces oxidative stress and promotes ROS generation in HEK293T and HK-2 cell lines. The elevated levels of lipid and protein oxidation, along with the upregulation of Nrf-2 and HMOX-1 mRNA expression, highlight the cellular response to counteract the oxidative damage caused by iron overload.

### 3.5 Iron overload activated molecular mechanism in the ferroptosis pathway

**FIGURE 5:**
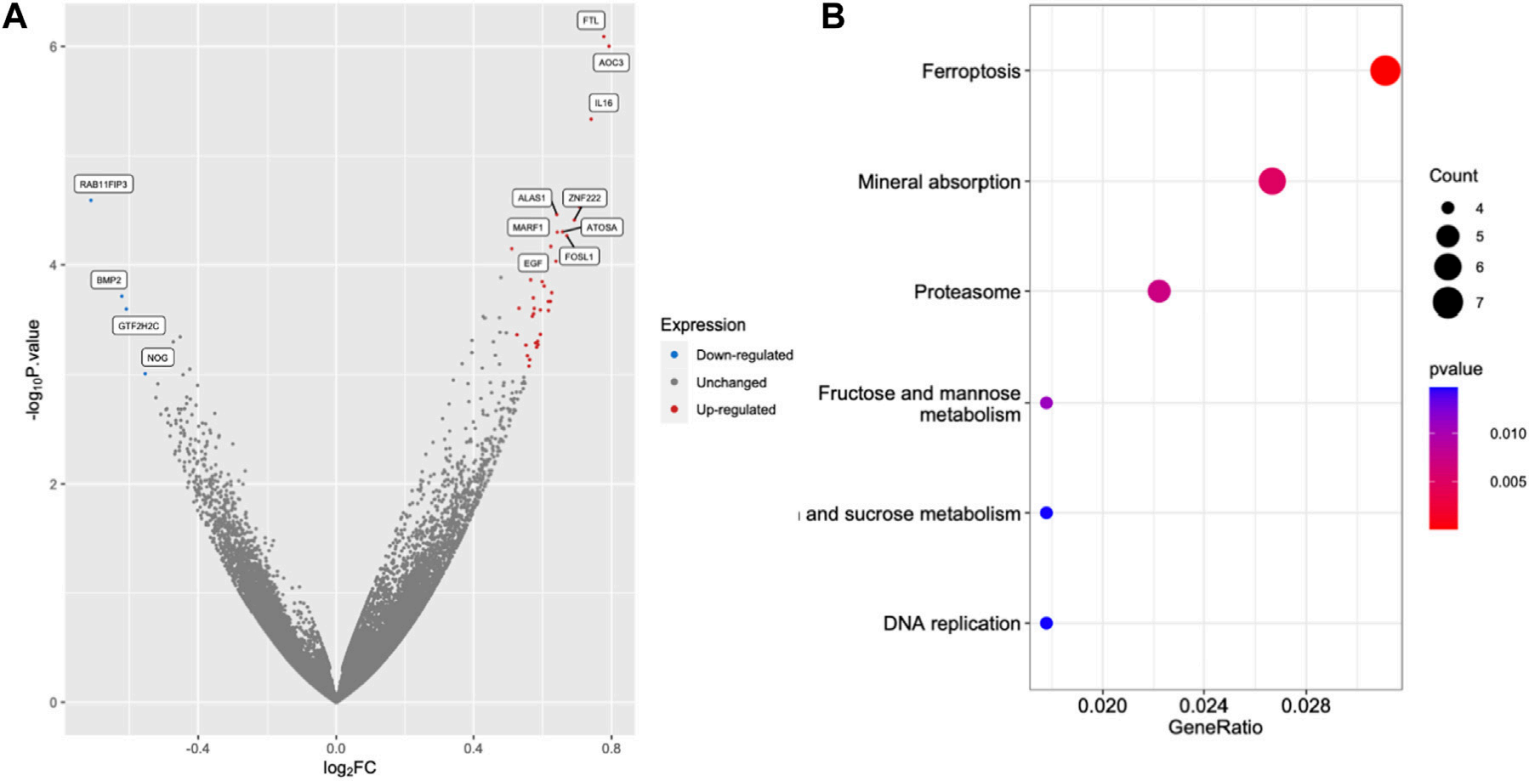
Excess iron activates the molecules in the ferroptosis pathway. The HEK293T cells were treated with 2.5 μM FAC and transcriptomic RNA sequencing was performed. **(A)** Volcano plot of genes based on the *p-value* and log_2_|FC|. The cut points of significant fold change (log_2_|FC| > 0.5) along with a statistically significant *p-value* < 0.001 were selected. Red and blue dots represented upregulated and downregulated genes, respectively. Grey dots represented unchanged genes. **(B)** Dot plots of the KEGG terms in the KEGG enrichment analysis. (FAC: Ferric ammonium citrate, FC: Fold change).

**FIGURE 6:**
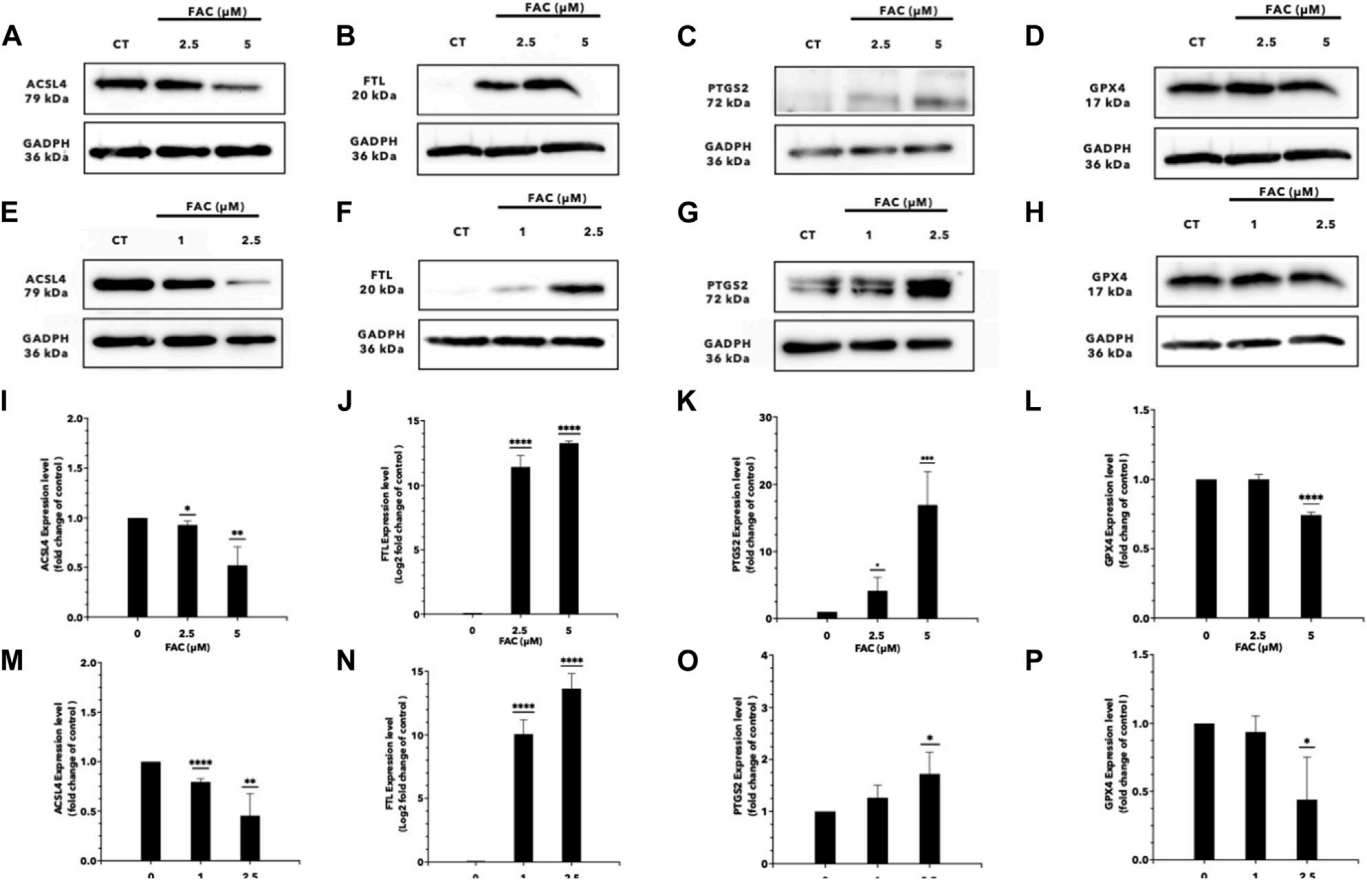
Iron overload affects the expression of the genes associated with ferroptosis. Protein expression of ferroptosis markers after FAC treatment was displayed with those of the control as Western blot band intensity **(A–H)** along with the corresponding graph of expression **(I–P)**. **(A,I)** ACSL4, **(B,J)** FTL, **(C,K)** PTGS2 and, **(D,L)** GPX4 expressions in HEK293T. **(E,M)** ACSL4, **(F,N)** FTL **(G,O)** PTGS2 and, **(H,P)** GPX4 expressions in HK-2 (^*^ *p* < 0.05,^**^ *p* < 0.01, ^***^ *p* < 0.001,^****^ *p* < 0.0001, ACSL4, Acyl-CoA Synthetase Long Chain Family Member 4; FTL, Ferritin light chain; PTGS2, Prostaglandin-endoperoxide synthase 2; GPX4, Glutathione peroxidase 4).

RNA sequencing (RNA-seq) was employed to explore the pathways related to iron toxicity. RNA was extracted from FAC-treated HEK293T cells, and transcriptome sequencing was performed (Figure 5). The most significantly upregulated gene was ferritin light chain (FTL) with a *p-value* of 8.13 × 10^−7^ and a log_2_|fold change| of 0.77 (Supplementary Table S2). We found that FAC treatment caused a significant increase in the ferroptosis pathway (KEGG metabolic pathways: hsa04216) (Supplementary Table S3). The expression of the key molecules in the ferroptosis pathway in the FAC-treated HEK293T and HK-2 cell lines was confirmed by real-time RT-PCR and Western blot analysis. The molecules examined were: FTL, prostaglandin-endoperoxide synthase 2 (PTGS2), acyl-CoA synthetase long-chain family member 4 (ACSL4), and glutathione peroxidase 4 (GPX4), which are known to play crucial roles in regulating ferroptosis (Figure 6 and Supplementary Figure S3). Our results revealed that iron overload induced significant upregulation of FTL and PTGS2 expression in both HEK293T and HK-2 cells, suggesting their potential involvement in iron-induced ferroptosis. In contrast, iron overload induced significant downregulation of ACSL4 and GPX4 expression in these cells, suggesting a disruption in lipid homeostasis and a reduction in antioxidant defense mechanisms, which may contribute to the progression of iron-induced ferroptosis.

### 3.6 Deferoxamine protected iron toxicity in kidney cell lines

**FIGURE 7:**
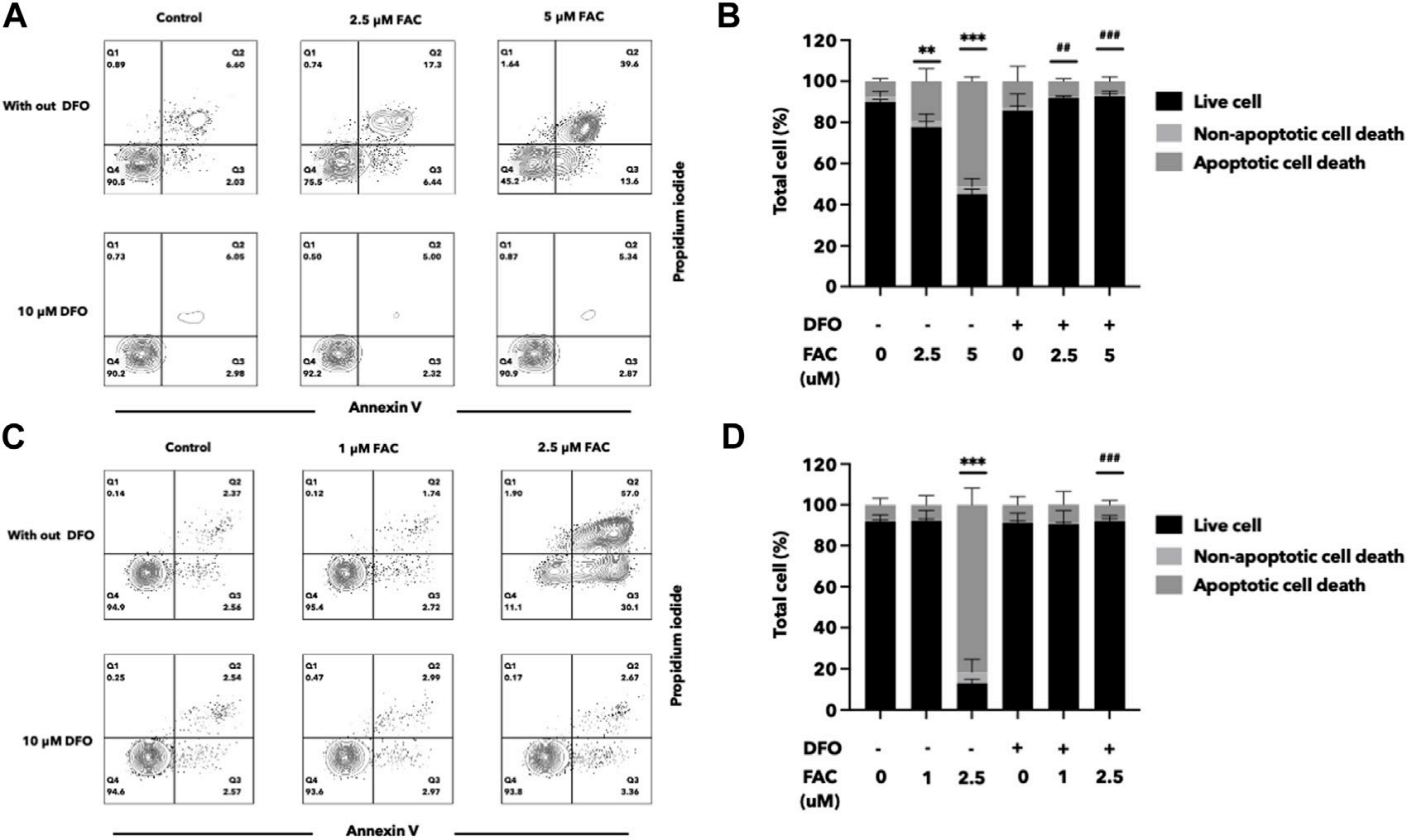
Deferoxamine can protect HEK293T and HK-2 cells against iron toxicity. The pre-treatment of DFO reduced cell death after FAC treatment, as observed by annexin V and propidium iodide staining. The results were shown in four quartiles with the percentage of the cell number. **(A)** HEK293T cells treated with 0, 2.5, and 5 μM of FAC with and without DFO pre-treatment. **(B)** HK-2 cells treated with 0, 1, and 2.5 μM of FAC with and without DFO pre-treatment. The corresponding graph shows the percentage of cell count in each quartile of the HEK293T **(C)** and HK-2 cells **(D)**. (FAC, Ferric ammonium citrate; DFO, Deferoxamine, ^**^ *p* < 0.01 when compared to the control group, ^***^ *p* < 0.001 when compared to the control group, ^##^ *p* < 0.01 when compared to the corresponding groups without DFO pre-treatment, ^###^ *p* < 0.01 when compared to the corresponding groups without DFO pre-treatment).

Deferoxamine (DFO) is an iron chelator commonly used in the treatment of iron overload disorders. To assess the efficacy of DFO in protecting the kidney cells against iron toxicity, HEK293T and HK-2 cells were exposed to 10 µM DFO for 2 h before or after treated with iron overload conditions. We evaluated the cell viability and cellular damage markers to determine the protective effects of DFO. The results demonstrated that iron overload significantly reduced cell viability in both HEK293T and HK-2 cell lines, but iron toxicity to the cells was significantly reduced both by pre-treatment or post-treatment with DFO (Figure 7; Supplementary Figure S4).

### 3.7 Urinary ferritin light chain level correlated with kidney function

**FIGURE 8:**
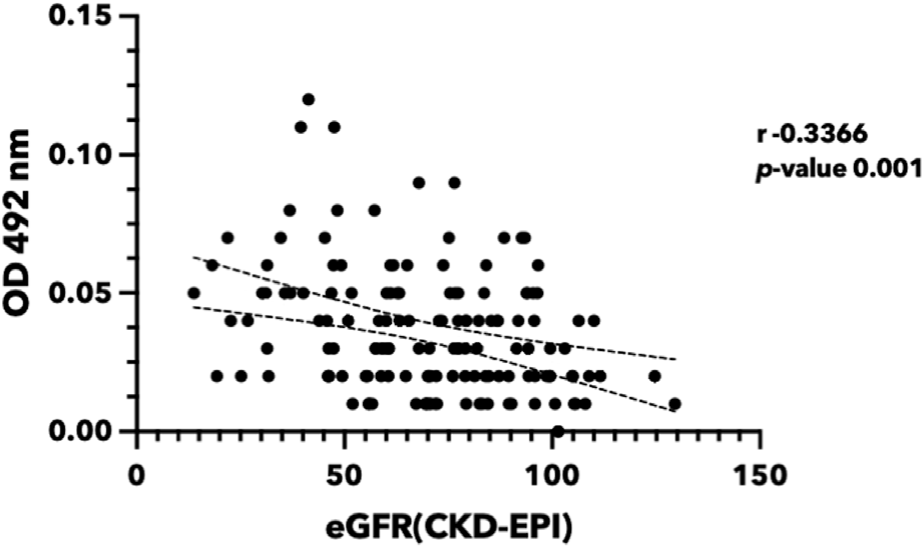
Urinary ferritin light chain level reversely correlated with renal function. Urine was collected from the participants in Khon Kaen Province, Thailand. eGFR was calculated using the CKD-EPI equation (Levey et al., 2009). FTL protein was quantified using indirect ELISA. (eGFR, estimated glomerular filtration rate; FTL, Ferritin light chain; ELISA, Enzyme-linked immunosorbent assay).

The presence of FTL in urine may provide conclusive evidence for the hypothesis that iron causes renal cell damage and increases FTL expression. In this study, we investigated the potential of FTL in urine samples as a biomarker to detect iron toxicity (Figure 8). Urine from volunteers with various eGFR was selected. FTL levels in urine were quantified using an indirect enzyme-linked immunosorbent assay (ELISA). The results revealed a significant elevation of FTL levels in the urine of the participants with low eGFR compared to that of the healthy volunteers. This increase in FTL levels indicates the presence of abnormal iron metabolism in the kidney.

## 4 Discussion

Iron, an essential mineral for various biological processes in the human body, plays a crucial role in functions such as oxygen transport, energy production, and DNA synthesis (MacKenzie et al., 2008; Fernandez-Garcia et al., 2022). Under normal conditions, the body maintains a balance between iron absorption, recycling and excretion. However, excessive iron accumulation, known as iron overload, results in an excess of iron accumulates in various organs (Kohgo et al., 2008; Pang et al., 2023; Zekavat et al., 2023). The kidney is one of the target organs of excess iron, leading to impairment of kidney function and damage (Zhang et al., 2022). Conversely, CKD status disrupts iron homeostasis due to increased losses, decreased iron absorption, increased storage, and decreased mobilization (Wish et al., 2018; Aranda-Rivera et al., 2021). The precise mechanisms through which iron induces kidney deterioration or destruction remain unclear.

Previous studies have reported a correlation between renal function and iron levels (Bross et al., 2009; Martines et al., 2013; Zhu et al., 2019; Zhang et al., 2022) similar to the correlation pattern observed between iron quantity and renal function in this study. The abnormal serum ferritin poses an elevated risk of kidney disease progression in Japanese patients (Fujisawa et al., 2023). Urinary iron excretion is associated with renal oxidative stress (Nakatani et al., 2018). To investigate the mechanisms of iron toxicity on renal cells, we employed kidney cell lines HEK293T and HK-2 as representative models of the kidney and evaluated the toxicity of FAC on these cells. Excess iron within the cells participates in Fenton’s reaction, reacting with hydrogen peroxide and generating free radicals or reactive oxygen species (ROS). The observation of Nrf2 activation in CKD supports this finding (Aranda-Rivera et al., 2022). These free radicals cause further damage to proteins and lipids within the cells. The results of this study demonstrated an increase in protein oxidation and carbonylation in FAC-treated kidney cells. Notably, iron also promoted lipid peroxidation, as evidenced by elevated levels of malondialdehyde (MDA), a byproduct of fatty acid peroxidation (Roch et al., 2022). These results are consistent with previously reported *in vivo* experiments (Annamalai et al., 2020). Thus, excess iron induces abnormal protein and lipid production in kidney cells. Lipid peroxidation and protein carbonylation are also hallmarks of ferroptosis.

The generation of free radicals further influenced cellular morphology and mitochondrial structure in the kidney cells. A previous *in vivo* study demonstrated that iron accumulates in kidney cells (Annamalai et al., 2020) using TEM analysis, which revealed cell membrane rupture and altered cell shape of the iron-treated cells. Moreover, mitochondrial morphology was disrupted with a decrease in cristae density. Intracellular iron accumulation was also observed in our study. These findings suggest that iron-induced free radicals affect cell and mitochondrial morphology and contribute to apoptosis. The interplay between ferroptosis and mitochondrial damage in other diseases has been reported (Neitemeier et al., 2017), and the role oxidative stress plays in mitochondrial changes has been reviewed (Aranda-Rivera et al., 2021). However, further investigations are needed to elucidate the underlying molecular mechanisms involved in these morphological changes and their impact on kidney cell function and viability (Bernardi et al., 2023).

In this study, flow cytometry analysis using Annexin V/PI staining confirmed apoptosis-induced cell death by excess iron. Notably, transcriptome investigation of iron-treated renal cells through RNA sequencing revealed the involvement of the ferroptosis pathway in iron-induced kidney cell death. Among the examined representative markers of ferroptosis, FTL and PTGS2 were overexpressed, the expression of the key antioxidant enzyme GPX4 decreased, and ACSL4 expression was suppressed. ACSL4 plays a critical role in ferroptosis by modulating the composition of cell membrane lipids, making them more susceptible to peroxidation and subsequent cell death (Yang et al., 2022). Therefore, further understanding the relationship between ACSL4 and ferroptosis is essential to unravel the mechanisms underlying this unique form of regulated cell death, and may have implications for developing therapeutic strategies targeting ferroptosis-related diseases, such as certain cancers and neurodegenerative disorders (Sun et al., 2022). In this regard, the toxic effects induced by iron were effectively protected by both pre and post-treatment of the kidney cells with deferoxamine (DFO), an iron-chelating agent. In the case of pre-treatment, administering DFO before exposure to FAC establishes a chelating environment that mitigates the impact of excess iron, potentially preventing or reducing cellular damage. Post-treatment, on the other hand, involves administering DFO after exposure to FAC. In this scenario, DFO continues to chelate excess iron, minimizing the ongoing detrimental effects of iron toxicity on the cells.

Ferritin, composed of heavy (FTH) and light (FTL) chains, forms a protein shell that stores excess iron in a non-toxic and soluble form (39). Ferritin plays many roles in sensing and regulating cellular iron levels (Carmona et al., 2014). We demonstrated that iron induces kidney cell death, with a subsequent increase in FTL within the cells. When cellular iron levels rise, ferritin molecules capture and store excess iron, preventing iron-induced oxidative damage. Recent research using a CKD mice model showed that selective myeloid *Fth1* knockout caused a reduction in renal fibrosis compared to the wild-type control (Patino et al., 2023), suggesting the crucial role of ferritin in controlling iron in CKD patients. Although numerous investigations sought out biomarkers involved in iron metabolism in the kidney, none found a correlation with iron-induced kidney damage (Bahr et al., 2019; Tomasz et al., 2021; Zapora-Kurel and Malyszko, 2021). Urine-based FTL detection is a non-invasive and convenient approach for assessing kidney damage in patients that serves as an alternative to traditional blood-based biomarkers. In our investigation, we noted a slight association between serum ferritin and kidney function (Supplementary Figure S2B). In contrast, urine ferritin exhibited a more robust correlation with eGFR. Although the potential for elevated serum ferritin levels due to inflammation exists, we contend that the studied population, consisting of individuals from villages, is not predominantly influenced by any suspected inflammatory conditions. However, further research is needed to use larger sample numbers and to explore its potential applications in various clinical settings to validate the diagnostic accuracy and clinical utility of urinary FTL as a screening tool for kidney iron toxicity.

**FIGURE 9:**
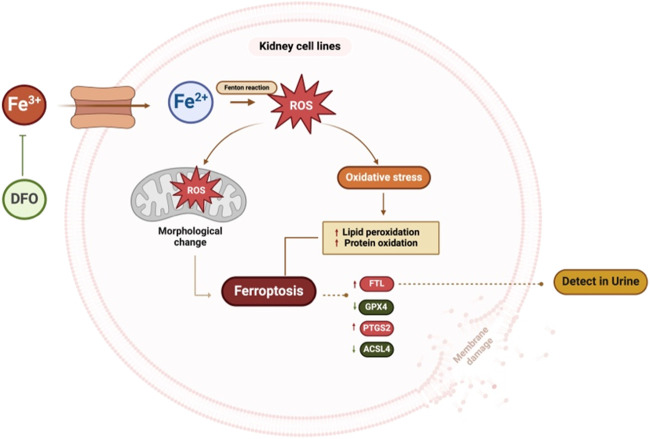
Summary figure of this study. Kidney cells treated with excess iron showed an increase in ROS and mitochondrial morphological changes, leading to cell death. Excess iron activates the ferroptosis pathway, resulting in an increase in the expression of ferroptosis markers, FTL and PTGS2, and a decrease in the expression of ACSL4 and GPX4. These findings suggest that excess iron disrupts cellular balance, affecting key proteins and potentially contributing to kidney cell death. Pre-treatment with DFO inhibits iron-induced cell death. (ROS: Reactive oxygen species, FTL: Ferritin light chain, PTGS2: Prostaglandin-endoperoxide synthase 2, ACSL4: Acyl-CoA Synthetase Long Chain Family Member 4, GPX4: Glutathione peroxidase 4, DFO: Deferoxamine). This figure was created with BioRender.com.

In conclusion, this is the first extensive study to explore the molecular mechanisms of iron-induced toxicity in kidney cells. The study highlights the involvement of the ferroptosis pathway and the modulation of key markers such as FTL, PTGS2, and GPX4 in iron-induced kidney cell death (Figure 9). Furthermore, we propose that urinary FTL can be a potential diagnostic biomarker for iron overload. These findings contribute to a better understanding of the molecular mechanisms underlying iron toxicity in kidney cells and suggest that the molecules in the ferroptosis pathway are potential therapeutic targets to mitigate iron-induced renal damage.

## Data Availability

The datasets presented in this study can be found in online repositories. The names of the repository/repositories and accession number(s) can be found below: https://www.ncbi.nlm.nih.gov/bioproject/PRJNA1049639.
