# Supplementary material for: Iron-induced kidney cell damage: insights into molecular mechanisms and potential diagnostic significance of urinary FTL
Source: Front Mol Biosci. 2024 Feb 21;11:1352032. doi: 10.3389/fmolb.2024.1352032 (PMC10916690; doi:10.3389/fmolb.2024.1352032)
Supplement: Supplementary file 2 [file Table2.DOCX]

**Supplementary Figure 1.** Dose response curve of kidney cell lines treated with ferric ammonium citrate (FAC) (A) HEK293T cell (B) HK-2 cell

**
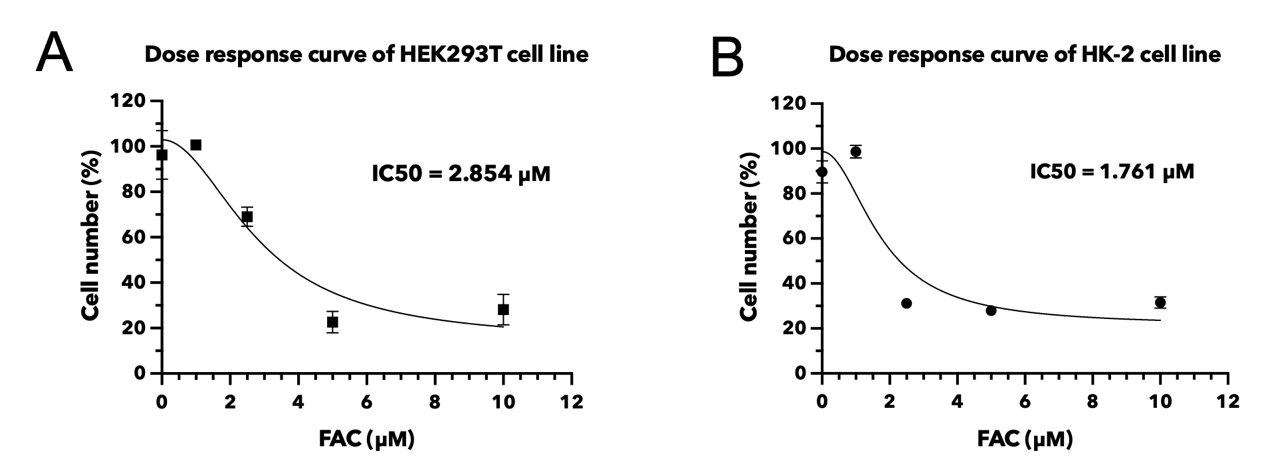
**

**Supplementary Figure 2.** mRNA expression of ferroptosis markers (A) FTL expression (B) PTGS2 expression (C) ACSL4 expression (D) HMOX-1 expression (**p* < 0.05,***p* < 0.01, ****p* < 0.001,*****p* <0.0001)

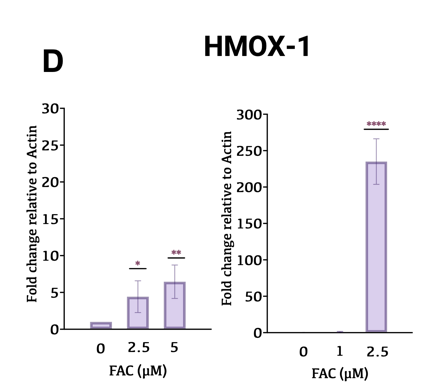


**Supplementary Table 1. Top 10 genes that had the lowest p-value.**

HEK293T cell treated with 2.5 μM of ferric ammonium citrate was compared to the control. Total RNA was performed transcriptome sequencing.

| Gene ID | Gene symbol | log2(FC) | P-value | P-adj |
| --- | --- | --- | --- | --- |
| 2512 | FTL | 0.77770662 | 8.13E-07 | 0.00741609 |
| 8639 | AOC3 | 0.79274028 | 9.96E-07 | 0.00741609 |
| 3603 | IL16 | 0.74068618 | 4.63E-06 | 0.02299389 |
| 9727 | RAB11FIP3 | -0.7115748 | 2.55E-05 | 0.08058152 |
| 105379045 | LOC105379045 | 0.70678809 | 2.93E-05 | 0.08058152 |
| 211 | ALAS1 | 0.64072736 | 3.44E-05 | 0.08058152 |
| 7673 | ZNF222 | 0.69162002 | 3.86E-05 | 0.08058152 |
| 56204 | ATOSA | 0.65743001 | 4.96E-05 | 0.08058152 |
| 9665 | MARF1 | 0.64199193 | 4.99E-05 | 0.08058152 |
| 8061 | FOSL1 | 0.66967859 | 5.41E-05 | 0.08058152 |

**Supplementary Table 2. Pathway enrichment analysis using KEGG database in DAVID website.**

The differentially expressed genes were subsequently analyzed for pathway involvement using the KEGG database.

| ID | Description | GeneRatio | BgRatio | pvalue | p.adjust | qvalue | Count |
| --- | --- | --- | --- | --- | --- | --- | --- |
| hsa04216 | Ferroptosis | 7/225 | 41/8461 | 8.91E-05 | 0.01363282 | 0.0135062 | 7 |
| hsa04978 | Mineral absorption | 6/225 | 60/8461 | 5.00E-03 | 0.36614174 | 0.362741 | 6 |
| hsa03050 | Proteasome | 5/225 | 46/8461 | 7.18E-03 | 0.36614174 | 0.362741 | 5 |
| hsa00051 | Fructose and mannose metabolism | 4/225 | 33/8461 | 1.09E-02 | 0.37552309 | 0.3720353 | 4 |
| hsa00500 | Starch and sucrose metabolism | 4/225 | 36/8461 | 1.47E-02 | 0.37552309 | 0.3720353 | 4 |
| hsa03030 | DNA replication | 4/225 | 36/8461 | 1.47E-02 | 0.37552309 | 0.3720353 | 4 |
