# Supplementary material for: Iron-induced kidney cell damage: insights into molecular mechanisms and potential diagnostic significance of urinary FTL
Source: Front Mol Biosci. 2024 Feb 21;11:1352032. doi: 10.3389/fmolb.2024.1352032 (PMC10916690; doi:10.3389/fmolb.2024.1352032)
Supplement: Supplementary file 1 [file Table1.DOCX]

Dec 6, 2023

Dear Editor,

I would like to submit the manuscript entitled **“Iron-Induced Kidney Cell Damage: Insights into Molecular Mechanisms and Potential Diagnostic Significance of Urinary FTL”** by Soraya Punchai, *et al*.

The manuscript has been approved by all authors. The content of this manuscript has not been published previously and is not under consideration for publication elsewhere. The study protocol was approved by the Human Research Ethics Committee, Khon Kaen University (HE661039 and HE651422). The English presentation of this manuscript was edited by Prof. Yukifumi Nawa, Editor-in-Chief, Parasitology International via the Faculty of Medicine Publication Clinic, KKU. The authors declare that there is no conflict of interest regarding the publication of this paper, and all authors have read and approved the submitted manuscript.

Acute and chronic renal disorders are important public health problems that harm and damage kidney cells, affecting millions of individuals globally. Iron overload can lead to the abnormal deposition of iron in organs, particularly the kidneys, resulting in tissue damage and the development of kidney diseases. There is an urgent need to comprehend the mechanisms of iron-induced kidney disease due to increasing concerns related to iron overload and its impact on renal health. The molecular mechanism of iron-induced toxicity in kidney cells has never been thoroughly investigated before. We have verified the link between body iron levels and renal function. Ferroptosis is a recently identified form of regulated cell death involving iron-dependent lipid peroxidation. The study highlights the involvement of the ferroptosis pathway and the modulation of key markers such as FTL, PTGS2, and GPX4 in iron-induced kidney cell death. These results advance our knowledge of the molecular pathways driving iron toxicity in renal cells and offer possible diagnostic and treatment approaches, like the identification of urinary FTL.

We think this paper has important messages that will interest scientists, biochemists, and all readers of the **Frontiers in Molecular Biosciences**. We would be grateful if the manuscript could be reviewed and considered for publication as an **original article** in this journal.

Sincerely yours,

Worachart Lert-itthiporn, MD., Ph.D.

Department of Biochemistry, Faculty of Medicine, Khon Kaen University,

123 Mittaparb road, Muang, Khon Kaen 40002, Thailand

Tel/Fax: +66-4334-8386

e-mail: woracle@kku.ac.th
